# Plakoglobin: Role in Tumorigenesis and Metastasis

**DOI:** 10.1155/2012/189521

**Published:** 2012-03-08

**Authors:** Zackie Aktary, Manijeh Pasdar

**Affiliations:** Department of Cell Biology, University of Alberta, Edmonton, AB, Canada T6G 2H7

## Abstract

Plakoglobin (**γ**-catenin) is a member of the Armadillo family of proteins and a homolog of **β**-catenin. As a component of both the adherens junctions and desmosomes, plakoglobin plays a pivotal role in the regulation of cell-cell adhesion. Furthermore, similar to **β**-catenin, plakoglobin is capable of participating in cell signaling. However, unlike **β**-catenin that has well-documented oncogenic potential through its involvement in the Wnt signaling pathway, plakoglobin generally acts as a tumor/metastasis suppressor. The exact roles that plakoglobin plays during tumorigenesis and metastasis are not clear; however, recent evidence suggests that it may regulate gene expression, cell proliferation, apoptosis, invasion, and migration. In this paper, we describe plakoglobin, its discovery and characterization, its role in regulating cell-cell adhesion, and its signaling capabilities in regulation of tumorigenesis and metastasis.

## 1. Introduction

Plakoglobin (also known as *γ*-catenin) is a member of the Armadillo family of proteins and a structural and functional homolog of *β*-catenin. These catenin proteins have two major roles in the cell: the mediation of cell-cell adhesion and cell signaling. As adhesive proteins, both *β*-catenin and plakoglobin interact with the cytoplasmic domain of cadherins, thereby tethering the cadherin proteins to the cytoskeleton. In addition to their cell-cell adhesive functions, both *β*-catenin and plakoglobin interact with a number of intracellular partners including signaling proteins and transcription factors, which accounts for their involvement in cellular signaling [1–4]. Despite these similarities, a major difference between *β*-catenin and plakoglobin emerges when considering their signaling functions. While *β*-catenin has a well-defined oncogenic potential as the terminal component of the Wnt signaling pathway [5–7], plakoglobin is typically associated with tumor/metastasis suppressor activity [8–10]. However, the mechanisms that underlie this activity remain undefined. In this paper, we have focused on the potential roles of plakoglobin during tumorigenesis and metastasis in an attempt to define how this often overlooked protein contributes to these complex processes.

## 2. Plakoglobin: Initial Identification and Early Characterization

Plakoglobin was initially identified as an 83 kDa protein component of the desmosomal plaque [11]. Subsequently, using monoclonal antibodies, cDNA cloning, and a combination of biochemical, morphological, and molecular approaches, Cowin et al. [12] demonstrated that this 83 kDa protein was present in both desmosomes and the adherens junction and was given the name plakoglobin.

Although plakoglobin was identified as a junctional protein, the role that it played in these junctional complexes was unclear, and the partners with which plakoglobin interacted were not identified. It was not until several years later that coimmunoprecipitation experiments showed that plakoglobin interacted with the desmosomal cadherin desmoglein, thereby confirming plakoglobin as a constituent of the desmosomes [13]. In addition, several groups showed that E-cadherin (initially known as uvomorulin) immunoprecipitates contained three distinct proteins, which became known as *α*-, *β*-, and *γ*-catenin [14–16]. These studies showed that these three catenin proteins, with molecular weights of approximately 102, 88, and 80 kDa, respectively, interacted with the cytoplasmic domain of E-cadherin. Further work analyzing the formation and stability of the E-cadherin-catenin complexes suggested that the E-cadherin-*β*-catenin complex was formed immediately after E-cadherin synthesis and was very stable. Interestingly, these studies also found that *α*-catenin could not be found in association with E-cadherin independent of *β*-catenin, suggesting that *β*-catenin was a physical link between E-cadherin and *α*-catenin. However, since *γ*-catenin was found to be only loosely associated with E-cadherin, it was determined that the main adhesive complexes consisted of E-cadherin, *β*-catenin, and *α*-catenin, although the existence of a separate E-cadherin-*γ*-catenin complex could not be ruled out [16].

At this time there was some confusion as to the identity of the catenin proteins and their relationship to plakoglobin. It soon became evident that plakoglobin was a homolog of *β*-catenin, a 92 kDa E-cadherin-associated protein [17]. However, it was not until the work of Knudsen and Wheelock [18] that it became clear that the 80 kDa protein that was associated with E-cadherin was indeed plakoglobin. In this study, the authors showed that plakoglobin interacted with both E- and N-cadherin and that it was a distinct protein from *β*-catenin [18]. This finding was confirmed by work from other groups demonstrating that plakoglobin and *γ*-catenin were indeed the same protein [1, 19].

Subsequent analysis of the kinetics of plakoglobin synthesis and associations with cadherins demonstrated that following synthesis, plakoglobin interacted with both desmoglein and E-cadherin in both the soluble and cytoskeleton-associated pools of cellular proteins. In addition, a distinct, cadherin-independent pool of plakoglobin was also observed, suggesting that plakoglobin may have a role in the cell in addition to cell adhesion. Finally, phosphorylation experiments revealed that whereas the insoluble (cadherin-associated) pool of plakoglobin was serine phosphorylated, the soluble pool was serine, threonine, and tyrosine phosphorylated, suggesting that these different pools of plakoglobin are differentially regulated and perform varying functions [20]. Collectively, these studies demonstrated that plakoglobin is a homolog of *β*-catenin and a unique protein in that it is the only component common to both E-cadherin and desmosomal cadherin-containing junctions.

## 3. Plakoglobin Functions: Cell-Cell Adhesion

**Figure 1:**
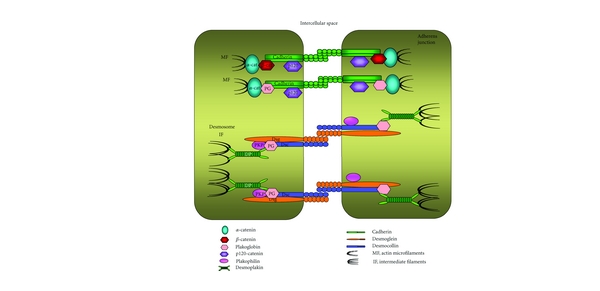
Cell adhesion complexes in epithelial cells. Cell-cell adhesion is maintained in epithelial tissues by the adherens junction and desmosomes. At the adherens junctions, E-cadherin forms extracellular interactions with E-cadherin molecules on neighboring cells. Intracellularly, E-cadherin interacts with either *β*-catenin or plakoglobin, which then interact with *α*-catenin, an actin-binding protein. A fourth catenin, p120-catenin, also interacts with E-cadherin and regulates its stability at the membrane. At the desmosome, the desmosomal cadherins (desmoglein and desmocollin) interact with plakoglobin and plakophilin, which interact with desmoplakin, which in turn associates with the intermediate filament cytoskeleton. The basic, core protein composition of the desmosomes is represented here: the exact protein constituents of the desmosomes and their interactions vary between different types of cells and tissues.

The most documented role of plakoglobin within the cell is in cell-cell adhesion. As such, plakoglobin is found in both adherens junctions and desmosomes (Figure 1). Adherens junctions are a ubiquitous type of intercellular adhesion structure present in both epithelial and nonepithelial cells, whereas desmosomes are adhesive junctions that confer tensile strength and resilience to cells and are present not only in epithelial cells but also in nonepithelial cells that endure mechanical stress, such as cardiac muscle. Both adherens junctions and desmosomes are cadherin based. Cadherins are single-pass transmembrane glycoproteins that form homotypic interactions with cadherin proteins on neighboring cells. Intracellularly, cadherins interact with proteins of the catenin family. At the adherens junction, the C-terminal domain of E-cadherin interacts, in a mutually exclusive manner, with *β*-catenin or plakoglobin, which then interacts with *α*-catenin, which is an actin-binding protein. A fourth catenin protein, p120-catenin, interacts with the juxtamembrane domain of E-cadherin and is important for E-cadherin dimerization and stability at the membrane (Figure 1; for reviews see [21, 22]). At the desmosome, the desmosomal cadherins (desmocollins and desmogleins) interact intracellularly with plakophilin and plakoglobin, which interact with desmoplakin, an intermediate filament binding protein (Figure 1; for reviews, see [23, 24]).

The identification of plakoglobin as a constituent of both the adherens junction and the desmosomes suggested that it might play an important role in regulating cell-cell adhesion. However, the observation that the adherens junctions could exist as a complex containing E-cadherin, *β*-catenin, and *α*-catenin, independent of plakoglobin [16] questioned the necessity of plakoglobin, at least at the adherens junctions. Regardless, it soon became apparent that plakoglobin does have an essential role in regulating cell-cell adhesion.

It had been previously shown that disruption of E-cadherin-based cell-cell adhesion led to a transformed and/or invasive phenotype while reexpression of E-cadherin in cells lacking its expression resulted in a mesenchymal to epithelial phenotypic transition [25–31]. Furthermore, reduced expression of E-cadherin was known to inversely correlate with the differentiation grade of tumors [32–37]. While it was clear that these E-cadherin-based junctions were important for the maintenance of an “epithelial” phenotype, the role of plakoglobin in this phenomenon was not discerned until it was shown that the expression of E- or P-cadherin alone in murine spindle cell carcinomas that lacked endogenous expression of these proteins was not sufficient to modify the morphology or tumorigenicity of these cells [38]. Although these cadherins were expressed in the cells, localized to the cell membrane, and interacted with both *α*- and *β*-catenin, they did not interact with plakoglobin. Further analysis showed that the levels of plakoglobin in these cells were very low, thus accounting for the absence of plakoglobin association with E-cadherin. From this work, the authors suggested that the association of the E-cadherin-catenin complex with plakoglobin may be necessary for its tumor suppressing activity.

Another significant role for plakoglobin in the regulation of cell-cell adhesion was discovered when studies showed that A431 epithelial cells treated with dexamethasone (which resulted in the isolation of fibroblastic A431 cells lacking E-cadherin but expressing desmoglein) were unable to form desmosomes upon exogenous expression of E- or P-cadherin, despite the formation of the adherens junction in these cells [39]. Interestingly, the authors observed that although plakoglobin was present at low levels in these cells, it was not coimmunoprecipitated with the exogenously expressed E-cadherin; in fact, the plakoglobin found in these cells coprecipitated with desmoglein. To examine the possibility that plakoglobin plays a regulatory role in desmosome formation, the authors expressed an E-cadherin-plakoglobin chimeric protein capable of forming stable adherens junctions in the cells and observed desmosome formation. While it had been previously observed that adherens junction formation not only preceded, but was also a prerequisite for desmosome formation [40–48], this was the first indication that plakoglobin served as a molecule involved in crosstalk between both junctional complexes in epithelia.

Following this study, our laboratory demonstrated the role of plakoglobin in adhesive junction formation by expressing low/physiological levels of plakoglobin in SCC9 cells, a squamous cell carcinoma cell line that lacks the expression of both plakoglobin and E-cadherin [9, 49]. Following exogenous plakoglobin expression, SCC9 cells underwent a mesenchymal to epidermoid phenotypic transition that was concurrent with the stabilization of N-cadherin and the formation of desmosomes and well-organized N-cadherin-containing adherens junctions [9]. This result confirmed that plakoglobin expression was necessary for desmosome formation and also demonstrated that plakoglobin-N-cadherin interactions could occur prior to desmosome formation. Other studies have further characterized the role of plakoglobin in desmosome assembly and function. Palka and Green [50] demonstrated the role of plakoglobin's C terminus for the proper assembly of the desmosomal plaque, and Acehan et al. showed that plakoglobin is essential for the efficient binding of desmoplakins to the intermediate filaments [51]. Furthermore, plakoglobin was shown to be necessary for the recruitment of plakophilin 3 to the membrane, desmosome formation, efficient cell-cell adhesion, and inhibition of cell migration and invasion [52, 53]. Finally, work from Birchmeier's laboratory showed that plakoglobin double knockout mice died during embryogenesis as a result of disrupted heart function due to the loss of stable desmosomes in the intercalated discs of cardiac muscle, further confirming the essential role of plakoglobin in desmosome formation and function [48, 54].

## 4. Plakoglobin Functions: Cell Signaling

### 4.1. Initial Observations and Controversy

The first clue that plakoglobin might participate in cell signaling came from studies of the exogenous expression of Wnt-1 in PC12 cells. In these cells, plakoglobin levels were increased, and it underwent membrane redistribution, suggesting that, in addition to *β*-catenin levels, Wnt-1 can modulate plakoglobin levels and localization [55]. Subsequently, Karnovsky and Klymkowsky [56] demonstrated plakoglobin signaling activity by microinjecting mRNAs-encoding plakoglobin into fertilized *Xenopus* embryos, resulting in dorsalized gastrulation and anterior axis duplication. In this study, the exogenously expressed plakoglobin localized both at the plasma membrane and in punctate nuclear aggregates. Furthermore, the coinjection of mRNAs-encoding plakoglobin as well as the cytoplasmic domain of desmoglein suppressed both dorsalized gastrulation and anterior axis duplication. In these embryos, plakoglobin was localized primarily to the plasma membrane with some perinuclear distribution. These results suggested that plakoglobin has signaling ability similar to *β*-catenin, but when it is sequestered at the plasma membrane (as part of desmosomes), plakoglobin is unable to participate in cell signaling.

This initial finding suggested that plakoglobin may have signaling functions similar to its homologs *β*-catenin and the *Drosophila *Armadillo protein. However, subsequent studies from various groups have demonstrated that while plakoglobin does indeed have signaling capabilities, it appears to function as a tumor suppressor rather than a tumor promoter. The first demonstration of this phenomenon occurred when Simcha et al. [8] found that plakoglobin expression in SV40-transformed NIH3T3 cells decreased the ability of these cells to form tumors in syngeneic mice. This growth suppressive effect of plakoglobin was augmented by cotransfection with N-cadherin. The authors also expressed plakoglobin in the renal carcinoma cell line KTCTL 60, which lacks endogenous expression of E-cadherin and desmosomal cadherins, *α*-catenin, *β*-catenin, plakoglobin, and desmoplakin and induces tumor formation in mice. Plakoglobin expression in KTCTL 60 cells also inhibited the tumorigenicity of these cells in syngeneic mice. Notably, the authors showed that the majority of the plakoglobin in these cells was Triton X-100 soluble, suggesting that it was not junction associated. This result was of significance because it demonstrated that plakoglobin could suppress tumor formation independent of its role in mediating cell-cell adhesion.

These studies made it clear that plakoglobin was capable of cell signaling and able to act as a tumor suppressor. Numerous subsequent studies have described the signaling function of plakoglobin as primarily one of tumor suppression, although a few reports have suggested that similar to *β*-catenin, plakoglobin may have oncogenic activity. In the following sections, we will present the experimental evidence for both the tumorigenic and tumor suppressive activities of plakoglobin and propose possible explanations for these observed discrepancies.

### 4.2. Plakoglobin Oncogenic Activity

Kolligs et al. [57] have shown that the tumor suppressor adenomatous polyposis coli (APC), which was already known to regulate the levels of *β*-catenin, could also regulate plakoglobin protein levels. In this study, the authors also showed that exogenous expression of plakoglobin in rat RK3E cells, which express considerable amounts of endogenous plakoglobin and *β*-catenin [57, 58], resulted in a transformed phenotype, which they suggested was dependent on the upregulation of the oncogene c-Myc and activation of Tcf/Lef signaling. More recently, Pan et al. [59] have shown that the exogenous expression of plakoglobin in HCT116 colon carcinoma cells, which express a mutant *β*-catenin protein that cannot be degraded [60], resulted in genomic instability and increased invasion and migration.

**Figure 2:**
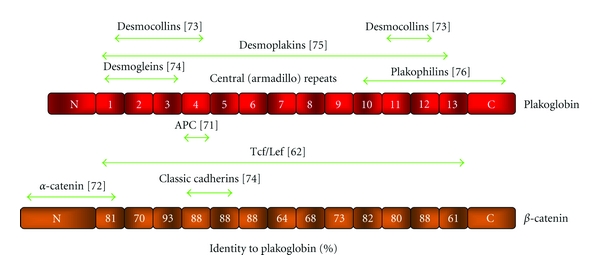
Schematic structure of *β*-catenin and plakoglobin. Both *β*-catenin and plakoglobin contain 13 Armadillo repeats that are flanked by N- and C-terminal domains, respectively. The degree of homology between *β*-catenin and plakoglobin for each Armadillo domain is indicated. Protein partners that interact with plakoglobin and the domains involved in these interactions are indicated. The corresponding references are listed in brackets (see [71–76]).

Both of these studies concluded that plakoglobin possessed oncogenic activity. However, it must be noted that several lines of evidence suggest that the oncogenic activity of plakoglobin may be indirect and achieved through modulation of the protein levels and signaling ability of *β*-catenin [61–67]. Since plakoglobin and *β*-catenin interact with some of the same proteins and display high sequence homology (Figure 2, [2, 4, 68, 69]), it became evident that plakoglobin may, in fact, be able to promote tumorigenesis by interacting with proteins that would normally sequester *β*-catenin (e.g., E-cadherin, Axin, APC), which would result in increased levels of cytoplasmic and nuclear *β*-catenin and in turn enhanced signaling. Indeed, following the observation that plakoglobin expression resulted in *Xenopus *axis duplication [56], the same group showed that this outcome did not depend on the nuclear localization of plakoglobin, since membrane-anchored forms of this protein produced the same axis duplication [70]. This demonstrated that nuclear plakoglobin was inconsequential in inducing a Wnt-like phenotype, since the cytoplasmic plakoglobin induced this same phenotype. At the same time, Salomon et al. [61] showed that overexpression of plakoglobin resulted in the displacement of *β*-catenin from, and the increased association of plakoglobin with, the N-cadherin-containing adherens junctions. Furthermore, excess cytoplasmic *β*-catenin was able to translocate into the nucleus. This was supported by other work, which showed that overexpression of plakoglobin in NIH3T3 cells resulted in the nuclear accumulation of *β*-catenin and that overexpression of the Wnt coactivator Lef-1 in MDCK cells resulted in its preferential interaction with *β*-catenin (instead of plakoglobin). Subsequently, the *β*-catenin-Lef complexes were localized to the nucleus [62], suggesting that when both plakoglobin and *β*-catenin were present within the cell, *β*-catenin-Lef complexes were more readily formed and transcriptionally active. Further examination of the ability of plakoglobin to signal via interactions with the Tcf/Lef family of transcription factors showed that although plakoglobin interacted with Lef-1, this complex was inefficient in binding to DNA, whereas *β*-catenin-Lef-1 complexes more readily bound DNA [63]. This study also demonstrated that overexpression of plakoglobin resulted in increased *β*-catenin-Lef-1 complex formation and its association with DNA. Further analysis of the transactivation potential of *β*-catenin and plakoglobin demonstrated that *β*-catenin was a much stronger activator of Tcf/Lef target genes than plakoglobin [64].

As mentioned earlier, we have previously shown that the expression of low/physiological levels of plakoglobin in plakoglobin-deficient SCC9 cells induced a mesenchymal to epidermoid change in phenotype, whereas its overexpression resulted in foci formation and decreased apoptosis, which was concurrent with the upregulation of the prosurvival protein Bcl-2 [77]. Using cDNAs-encoding plakoglobin fused to nuclear localization or nuclear export signals (NLS and NES), we subsequently showed that Bcl-2 levels were upregulated in plakoglobin overexpressing SCC9 cells regardless of plakoglobin localization. Furthermore, in these cells, *β*-catenin-N-cadherin interactions were decreased, and *β*-catenin accumulated in the nucleus, interacted with Tcf, and its signaling was increased [65], confirming that the overexpressed plakoglobin acted indirectly by enhancing the signaling capability of *β*-catenin.

The above studies describing the oncogenic potential of plakoglobin may also be as a result of *β*-catenin. In Kolligs's study [57] where plakoglobin was overexpressed in RK3E cells (which express endogenous *β*-catenin and plakoglobin [58]), it was not determined if plakoglobin could activate c-Myc expression in the absence of *β*-catenin or whether either of these catenins was detected in the nucleus in association with the c-Myc promoter. In addition, in Pan's study [59] in which HCT116 cells showed increased genomic instability and migration and invasion upon plakoglobin expression, the endogenous *β*-catenin was a mutant protein that was unable to be phosphorylated and subsequently degraded [60]. While much of the *β*-catenin localized to the membrane in these cells [78], plakoglobin expression most likely led to decreased *β*-catenin-cadherin interactions and increased *β*-catenin signaling. In support of this prediction, HCT116 cells overexpressing plakoglobin showed increased expression of the oncogenes securin and c-Myc and decreased expression of E-cadherin, all of which are documented *β*-catenin target genes [79–81]. Taken together, the evidence suggests that although plakoglobin expression may lead to a transformed phenotype, it is likely that this outcome is associated with increased oncogenic *β*-catenin signaling rather than oncogenic activity due directly to plakoglobin.

### 4.3. Plakoglobin Signaling in *β*-Catenin Null Cells

While the oncogenic signaling activity of plakoglobin discussed above can be attributed to the signaling activity of *β*-catenin rather than plakoglobin itself, this cannot account for all of the observations regarding plakoglobin signaling. Recent studies attempting to discern the signaling activity of plakoglobin independent of *β*-catenin have used tissue culture cell lines that lack the endogenous expression of *β*-catenin [82–85]. These studies have shown that in the absence of *β*-catenin, plakoglobin does indeed have Tcf/Lef-mediated transcriptional activity, although this activity is less than that of *β*-catenin-Tcf complexes. Interestingly, although these studies have demonstrated that plakoglobin can signal through forming transcriptional complexes with Tcf/Lef transcription factors, they did not assess the tumor-forming properties of these cells, so it remains unclear as to whether these cells possessed transformed or nontransformed properties. To that end, it has been demonstrated that plakoglobin or *β*-catenin expression in renal carcinomas lacking endogenous *β*-catenin and plakoglobin resulted in the upregulation of Nr-CAM, a neuronal cell adhesion molecule that can be regulated by both *β*-catenin and plakoglobin [82]. Furthermore, Nr-CAM expression in NIH3T3 cells conferred a more tumorigenic and invasive phenotype on these cells. Significantly however, although plakoglobin expression resulted in increased Nr-CAM levels in renal carcinomas and although plakoglobin-Tcf/Lef complexes can regulate Nr-CAM expression, the overall phenotype of these cells upon plakoglobin expression was nontumorigenic [8]. This showed that although plakoglobin may regulate *β*-catenin-target genes in the absence of *β*-catenin, it still may suppress tumorigenesis in the same cells. The homology between plakoglobin and *β*-catenin explains the ability of plakoglobin to signal through Tcf/Lef in the absence of *β*-catenin. Taken together, indeed, it is not surprising that if *β*-catenin is completely absent from a cell line, plakoglobin cannot only replace it in junctional complexes, but may also be able to regulate some *β*-catenin target genes (e.g., Survivin [85]). However, as a final note, it is important to consider that *β*-catenin-null tumors are extremely rare, and in most tumors and cell lines, plakoglobin signaling activity occurs in the presence of *β*-catenin.

### 4.4. Plakoglobin Tumor Suppressor Activity

Despite the observation that plakoglobin overexpression promotes tumorigenesis mediated by the oncogenic signaling of *β*-catenin, several studies examining the signaling function of plakoglobin have identified it as a tumor suppressor. We have previously shown that expression of physiological levels of plakoglobin in SCC9 cells, which lack endogenous plakoglobin and E-cadherin, resulted in a mesenchymal to epidermoid phenotypic transition, which was concurrent with the stabilization of N-cadherin, the formation of desmosomes, and the downregulation of *β*-catenin [9]. Furthermore, we have found that plakoglobin-expressing SCC9 cells showed a decreased growth rate compared to parental SCC9 cells. These results, taken together, demonstrated that not only could plakoglobin act as a tumor suppressor, but that potentially it does so by decreasing the levels of *β*-catenin.

The ability of plakoglobin to inhibit cell growth and proliferation was next observed when Charpentier et al. [10] expressed plakoglobin (under the control of an epidermal-specific promoter) in the basal cells of the epidermis as well as the hair follicles of transgenic mice. These authors showed that plakoglobin expression resulted in a reduced proliferative potential of the epidermal cells and that plakoglobin-expressing hair follicles had a significantly reduced growth phase, with hairs shorter by roughly 30% after plakoglobin expression.

Further evidence suggesting a growth suppressive activity for plakoglobin was provided in lung cancer, when it was shown that while *β*-catenin was uniformly expressed in various Nonsmall cell lung cancer (NSCLC) cell lines and lung primary tumors, plakoglobin expression was very low or completely absent [86]. The authors showed that exogenous expression of plakoglobin in the low-plakoglobin-expressing NSCLC cells resulted in decreased *β*-catenin-Tcf signaling, which was concurrent with decreased cell and anchorage-independent growth. This result further supported the idea that plakoglobin can act as a tumor suppressor by inhibiting the oncogenic activity of *β*-catenin.

Interestingly, when the authors treated these NSCLC cell lines with the DNA methylation inhibitor 5-aza-2′-deoxycytidine (AZA) or the histone deacetylase inhibitor trichostatin A (TSA), plakoglobin levels were increased. Previous analysis of the plakoglobin promoter had described CpG islands within the promoter [87], and while it had been observed that inhibition of DNA methylation could result in increased plakoglobin protein levels in at least one thyroid carcinoma cell line [88], this was the first indication that both DNA methylation and histone deacetylation played important roles in regulating plakoglobin expression.

The occurrence of methylated CpG islands within the plakoglobin promoter as well as histone deacetylation has not been limited to NSCLC cell lines. Various groups have shown that the plakoglobin promoter is methylated in prostate, bladder, trophoblastic, and mammary carcinomas [89–92], which is concurrent with a transformed phenotype. Canes et al. [90] have shown that treatment of bladder carcinoma cells with TSA resulted in increased plakoglobin expression and a decreased ability of these cells to form tumors in mice, once again suggesting a growth inhibitory activity of plakoglobin. Similarly, when mammary carcinoma cell lines were treated with AZA, increased plakoglobin levels were observed, as well as decreased soft agar colony formation and overall cell growth [92], indicative of decreased tumor-forming ability.

Several lines of evidence suggest that plakoglobin plays a role in regulating apoptosis, in addition to acting as a growth suppressor. In their work describing the effects of plakoglobin on hair growth in transgenic mice, Charpentier et al. [10] showed that plakoglobin expression decreased epithelial proliferation. Moreover, this expression also resulted in premature apoptosis, because TUNEL assays showed that the inner root sheath of the plakoglobin-expressing transgenic follicles underwent apoptosis two days earlier than in normal hair follicles. In agreement with these findings, we have previously shown that SCC9 cells expressing physiological levels of plakoglobin were more prone to undergo staurosporine-induced apoptosis when compared to parental SCC9 cells [77]. We have also observed that SCC9 cells expressing plakoglobin exclusively in the nucleus (SCC9-PG-NLS) showed decreased Bcl-2 levels compared to cells with overexpressed wild-type plakoglobin, which suggests that plakoglobin may play a more direct role in regulating the expression of apoptotic genes. More recently, it has been shown that mouse keratinocytes that lack endogenous plakoglobin expression are protected from etoposide-induced apoptosis, whereas plakoglobin-expressing keratinocytes readily undergo apoptosis upon etoposide treatment [93]. In this study, the authors demonstrated that plakoglobin-null keratinocytes were unable to release cytochrome c from the mitochondria and activate caspase 3, suggesting that plakoglobin plays a role in regulating the apoptotic cascade. Furthermore, the mRNA levels of the antiapoptotic protein Bcl-X_L_ were higher in the plakoglobin null keratinocytes, which could potentially have prevented the translocation of cytochrome c from the mitochondria. Finally, the expression of plakoglobin in the null keratinocytes resulted in decreased Bcl-X_L_ levels, caspase 3 activation, and apoptosis induction following etoposide treatment. Taken together, these studies have demonstrated that plakoglobin does have some role in apoptosis signaling and potentially may exert part of its tumor suppressor activity through the modulation of apoptosis.

### 4.5. Plakoglobin Metastasis Suppressor Activity

As the tumor suppressor activity of plakoglobin began to be revealed, it soon became evident that in addition to inhibiting the growth properties of carcinoma cell lines, plakoglobin also plays a role in regulating the invasive and migratory properties of cancer cells. The initial observation of plakoglobin metastasis suppressor activity was documented in human umbilical vascular endothelial cells (HUVEC), where plakoglobin was typically associated with sites of cell-cell contact [94]. Plakoglobin antisense oligonucleotides increased HUVEC migration, suggesting that the loss of plakoglobin expression led to an increased migratory phenotype. Concurrent with increased migration, the antisense treated HUVEC cells also became more prone to forming tubular structures in Matrigel, suggesting that plakoglobin knock down also promoted angiogenesis.

Mukhina et al. [95] further detailed the metastasis suppressor activity of plakoglobin using MCF-7 cells, which express membrane-localized E-cadherin and plakoglobin, and stable cell junctions. In this study, the authors treated MCF-7 cells with human growth hormone (hGH) and observed a downregulation of plakoglobin, a cytoplasmic distribution of E-cadherin and an increased migratory and invasive phenotype, which was accompanied by an increase in matrix metalloproteinase levels. Furthermore, the authors demonstrated that hGH-mediated invasiveness was dependent on Src kinase and also showed that chemical inhibitors of Src resulted in increased plakoglobin levels and, in turn, decreased invasion and migration. To discern the specific role of plakoglobin in these processes, the authors expressed plakoglobin in the hGH-treated MCF-7 cells, which resulted in both the decreased migration and invasiveness of these cells [95].

The metastasis suppressor activity of plakoglobin has also been described in bladder carcinomas, where the expression of plakoglobin in plakoglobin null cell lines resulted not only in decreased growth and tumorigenicity (as assessed by colony formation in soft agar and tumor formation in nude mice, resp.), but also in decreased invasive and migratory capabilities of the transfectants [96]. Similarly, knock down of plakoglobin using siRNAs resulted in the increased tumorigenic and invasive properties of bladder carcinoma cells relative to their plakoglobin-expressing parental cell lines. This study further demonstrated that plakoglobin expression did not affect Wnt/*β*-catenin signaling in these bladder carcinomas, which suggested that plakoglobin possessed tumor and metastasis suppressor activities independent of *β*-catenin.

The ability of plakoglobin to act as a metastasis suppressor independent of its role in cell-cell adhesion has been demonstrated using plakoglobin null keratinocytes [97], which were less adherent to one another and more migratory (as assessed by transwell migration assays). However, when wild-type plakoglobin was expressed in these cells, they became more adherent and less migratory. Using colloidal gold-coated coverslips, the authors were able to assess the migratory abilities of individual cells and observed that individual plakoglobin null keratinocytes were more migratory than their plakoglobin-expressing counterparts. The authors also showed that plakoglobin may regulate single keratinocyte migration by inhibition of Src signaling, which had been previously shown to promote migration and invasion of mammary carcinomas by downregulation of plakoglobin (see above [95]). These results suggested that plakoglobin could suppress migration through the modulation of cell-cell adhesion, as had been previously suggested. However, to determine whether plakoglobin could have an effect in migration independent of its role in cell-cell adhesion, plakoglobin null keratinocytes were transfected with cDNAs encoding mutant plakoglobin, missing either its N- or C-terminus (*α*-catenin binding and transactivation domain, resp.). The expression of either of these mutant proteins resulted in increased keratinocyte adhesiveness when compared to the plakoglobin null cells, demonstrating that these domains were dispensable for the adhesive function of plakoglobin. Importantly, the authors showed that whereas individual keratinocytes expressing the N-terminal-deleted plakoglobin were not migratory, those that expressed the C-terminal-deleted plakoglobin were migratory. This showed that plakoglobin could indeed suppress migration independent of its adhesive function (since keratinocytes expressing C-terminal-deleted plakoglobin were as adhesive to one another as wild-type plakoglobin expressing keratinocytes). Subsequent work using these plakoglobin null keratinocytes has suggested that plakoglobin affected individual cell motility by regulating the deposition of the extracellular matrix (ECM) protein fibronectin, actin cytoskeleton organization (which in turn regulates Src signaling), and RhoGTPases [98]. Collectively, these observations clearly demonstrate tumor/metastasis suppressor activity of plakoglobin independent of its role in cell-to-cell adhesion.

## 5. Plakoglobin Functions: Regulation of Gene Expression

When discussing roles for plakoglobin during tumorigenesis and metastasis, it is important to consider that while plakoglobin may function as both a regulator of cell-cell adhesion and an intracellular signaling molecule, it may also play a more active role in these processes through the regulation of gene expression. Evidence supporting the plakoglobin-mediated regulation of gene expression has started to emerge, and work from several groups, including ours, has suggested that plakoglobin can regulate the expression of genes involved in cell-cycle control, apoptosis, cell proliferation, and invasion.

Williamson et al. [99] have shown that plakoglobin acts as a repressor of the c-Myc gene. Using mouse keratinocytes and reporter assays, the authors of this study showed that plakoglobin suppressed c-Myc expression in a Lef-1-dependent manner, suggesting that when plakoglobin interacted with Lef-1, this complex was unable to promote gene expression. These findings confirmed previous results demonstrating the inefficiency of these complexes in binding DNA [62–64, 100]. This study further showed that the plakoglobin-mediated suppression was similar in both wild-type and *β*-catenin null keratinocytes, demonstrating that plakoglobin could regulate gene expression independent of *β*-catenin. Finally, using chromatin immunoprecipitation with plakoglobin antibodies, the authors demonstrated that plakoglobin and Lef-1 associated with the c-Myc promoter in keratinocytes undergoing growth arrest, which implicated the downregulation of c-Myc gene expression as a possible reason for the suppression of cell growth by plakoglobin.

Plakoglobin-mediated regulation of gene expression has also been shown in renal carcinoma cells. Shtutman et al. [101] found that the exogenous expression of plakoglobin in cells lacking both *β*-catenin and plakoglobin resulted in the increased expression of the tumor suppressor gene PML, a nuclear protein that forms nuclear bodies and is involved in the regulation of p53 activity. Importantly, the increased PML levels due to plakoglobin expression were independent of *β*-catenin and Tcf, since *β*-catenin was not detected in the plakoglobin-expressing cells and the deletion of Tcf/Lef sites in the PML promoter did not affect the ability of plakoglobin to increase PML gene expression. Together, these observations suggest that plakoglobin may regulate gene expression independent of Tcf/Lef.

In *β*-catenin null mesothelioma and colon carcinoma cells, Wnt3a stimulation led to the nuclear accumulation of plakoglobin and induced the expression of the antiapoptotic gene Survivin [85]. Coimmunoprecipitation and chromatin immunoprecipitation showed that plakoglobin formed a transcriptional complex with both Tcf and the histone acetyltransferase CBP and that this complex was associated with the Survivin promoter [85]. While this study clearly demonstrated that plakoglobin was capable of regulating *β*-catenin target genes in a *β*-catenin null background, it is again of importance to emphasize that *β*-catenin null tumors are very rare and that the plakoglobin-mediated regulation of gene expression occurs mainly in the presence of cellular *β*-catenin.

As previously discussed, Todorović et al. [98] have shown that plakoglobin can regulate cell motility by regulating Fibronectin and Rho-dependent Src signaling. This study also demonstrated that plakoglobin expression resulted in increased levels of Fibronectin mRNA without increasing expression from the Fibronectin promoter. However, by using Actinomycin D to inhibit transcription, the authors were able to demonstrate that plakoglobin expression led to the increased stability of Fibronectin mRNA, suggesting that in addition to its role in regulating gene expression at the level of transcription, plakoglobin may also regulate gene expression posttranscriptionally. However, the mechanisms underlying this action remain unclear. Overall, these studies suggest that plakoglobin regulates gene expression at the transcriptional and potentially at posttranscriptional levels.

## 6. Plakoglobin Expression in Human Tumors

The initial characterization of *JUP*, the gene encoding plakoglobin, mapped the gene to chromosome 17q21, proximal to the *BRCA1* gene [102]. In this study, the authors also analyzed RNA isolated from ovarian and breast cancer tumors and showed that loss of heterozygosity in these tumors and low-frequency mutations in the plakoglobin gene predisposed patients to familial breast and ovarian cancer. Since then, several groups have observed the loss of plakoglobin expression in a wide range of tumors, with the majority of these studies examining plakoglobin in conjunction with other adhesive junctional proteins. These studies have demonstrated that loss of plakoglobin expression in conjunction with the lack of expression of other cell-cell adhesion proteins such as E-cadherin, *α*-catenin, *β*-catenin, desmoglein, or desmoplakin resulted in increased tumor formation and size and was correlated with increased tumor stage, poor patient survival, and increased metastasis in bladder, pituitary, oral, pharyngeal, skin, prostate, and NSCLC tumors [103–111]. However, several studies have found that decreased levels of plakoglobin alone also occur in various tumors.

The loss of plakoglobin expression has been observed in melanocytic and thyroid tumors [112, 113]. Cerrato et al. [113] found that nearly 90% of papillary and follicular tumors showed decreased or loss of membrane plakoglobin localization. Decreased expression of the plakoglobin gene was also observed in prostate tumors, where methylation of the plakoglobin gene is prevalent in localized prostate cancer when compared to benign prostatic hyperplasia, suggesting that loss of plakoglobin expression was an early step in prostate tumorigenesis [89]. In oropharynx squamous cell carcinomas, decreased plakoglobin expression as well as its abnormal cytoplasmic distribution was correlated with increased tumor size and poor clinical outcome [114].

In colon carcinomas, Lifschitz-Mercer et al. [115] showed that *β*-catenin accumulated in the nuclei of cells of primary and metastatic adenocarcinoma and adenoma lesions, while the levels of nuclear plakoglobin were decreased in these tumors, suggesting that nuclear plakoglobin did not promote tumorigenesis in the colon. In esophageal cancers, while decreased levels of E-cadherin and plakoglobin were associated with poor differentiation and decreased patient survival, reduced plakoglobin levels alone correlated with lymph node metastasis [116]. The finding that reduced plakoglobin levels alone correlated with increased metastasis was not limited to esophageal tumors. In renal carcinomas, decreased plakoglobin levels have been associated with metastasis, and patients with tumors expressing plakoglobin showed significantly higher survival rates than those that did not [117]. Aberrant or decreased plakoglobin levels have also been reported in Wilms' tumors and soft tissue sarcomas, where the decrease in plakoglobin was associated with increased risk of pulmonary metastasis [118, 119]. In endometrial tumors, the aberrant expression of plakoglobin was correlated with myometrial invasion [120], whereas medulloblastoma tumors expressing plakoglobin were nonmetastatic, with no evidence of subarachnoid or hematogenous metastasis [121]. Finally, reduced plakoglobin expression was also correlated with increased lymph node metastasis in oral squamous cell and bladder tumors [122, 123]. Collectively, these observations suggest that lack or decreased expression of plakoglobin due to genetic or epigenetic causes in tumors of different origins is associated with poor clinical outcome and increased tumor formation and metastasis.

## 7. Growth/Metastasis Inhibitory Activities of Plakoglobin via Regulation of Gene Expression

We have developed two experimental model systems using squamous and breast carcinoma cell lines with no or very low plakoglobin expression and various degrees of transformation/invasiveness to specifically assess the growth/metastasis inhibitory activities of plakoglobin. Using a combination of molecular and cell biological approaches, including proteomics and transcriptome analysis, we compared the protein and mRNA profiles of plakoglobin-deficient and plakoglobin-expressing cell lines and their *in vitro* migration and invasiveness. These analyses led to the identification of several growth regulatory genes that were differentially expressed in plakoglobin-expressing transfectants compared to their plakoglobin-deficient parental cells.

**Figure 3:**
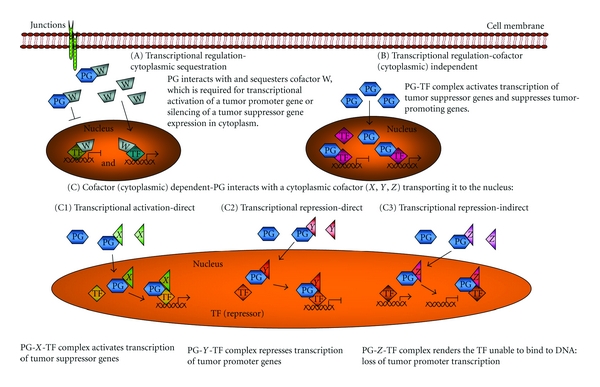
A potential model for regulation of gene expression by plakoglobin. Three concurrent mechanisms by which plakoglobin may regulate gene expression are proposed. (A) Cytoplasmic sequestration: plakoglobin sequesters a factor in the cytoplasm which, in the nucleus, suppresses the expression of a tumor suppressor gene or activates the expression of an oncogene. (B) Cytoplasmic cofactor independent: plakoglobin-transcription factor complexes promote the expression of tumor suppressor genes and repress the expression of oncogenes. (C) Cytoplasmic cofactor dependent: plakoglobin interacts with a cytoplasmic cofactor and this complex moves into the nucleus where it activates tumor suppressor gene expression or represses oncogenic gene expression. PG: plakoglobin; TF: transcription factor.

Comparison of the proteomic profiles of plakoglobin null SCC9 cells and their plakoglobin-expressing transfectants allowed us to identify several tumor/metastasis regulating proteins, which were differentially expressed in plakoglobin-expressing transfectants (SCC9-PG-WT) relative to parental SCC9 cells. We performed RNA microarray experiments to determine whether changes in gene expression upon plakoglobin expression accompanied these changes in protein levels and compared the transcriptome profiles of SCC9 cells and SCC9-PG-WT transfectants. Furthermore, to determine whether the subcellular distribution of plakoglobin had an effect on gene expression, we also compared the RNA profiles of SCC9 and SCC9-PG-WT cells with those of SCC9 cells transfected either with cDNAs-encoding plakoglobin fused with a nuclear localization signal (NLS) to express plakoglobin exclusively in the nucleus (SCC9-PG-NLS), or cDNAs-encoding plakoglobin fused with a nuclear export signal (NES) to express plakoglobin exclusively in the cytoplasm (SCC9-PG-NES). From these experiments, we identified three subsets of genes that were differentially expressed based on plakoglobin expression and its subcellular distribution: those whose differential expression required exclusively cytoplasmic plakoglobin, those whose differential expression required nuclear plakoglobin, and those whose differential expression required the ability of plakoglobin to shuttle between the nucleus and the cytoplasm. Based on the results of these experiments and analysis of the expression patterns of plakoglobin-target genes in relation to plakoglobin subcellular distribution, we propose that plakoglobin can regulate gene expression by three concurrent mechanisms (Figure 3).

The first of these mechanisms involves the action of plakoglobin in the cytoplasm, where it would sequester a protein involved in the regulation of gene expression. In this case, plakoglobin would prevent an inhibitor of a tumor suppressor gene or a promoter of an oncogenic gene from entering the nucleus and affecting gene expression. Plakoglobin target genes whose expression patterns were similar in SCC9-PG-WT and SCC9-PG-NES cells and were opposite to SCC9-PG-NLS cells would be considered part of this group.

The second mechanism involves nuclear localized plakoglobin, which would directly associate with a nuclear factor and regulate gene expression. In this case, plakoglobin would interact with a transcriptional activator and promote gene expression, or, conversely, it would interact with a transcriptional repressor and silence gene expression. Plakoglobin target genes whose expression patterns were similar in SCC9-PG-WT and SCC9-PG-NLS cells and were opposite to SCC9-PG-NES cells would be considered part of this group.

The vast majority of plakoglobin target genes, however, belonged to the third group of genes: those whose differential expression depended on the ability of plakoglobin to shuttle between the nucleus and the cytoplasm. In this case, plakoglobin would interact with some cytoplasmic cofactor, translocate into the nucleus, and regulate gene expression. Plakoglobin target genes whose expression patterns were similar in SCC9-PG-NES and SCC9-PG-NLS cells and were opposite to SCC9-PG-WT cells would be considered part of this group.

Following these proteomics and microarray analyses, we began our initial characterization of the regulation of potential target genes by plakoglobin. We have recently shown that plakoglobin expression in SCC9 cells resulted in the increased expression of the metastasis suppressors Nm23-H1 and -H2, both at the mRNA and protein levels [124]. Nm23 was the first metastasis suppressor identified, as it is often downregulated in metastatic tumors and its expression in invasive cell lines resulted in decreased migration and invasion (for review, see [125, 126]). We have observed that plakoglobin interacted with Nm23-H1 and -H2 in squamous cell, mammary, renal, and colon epithelial cell lines with the colocalization of these two proteins at sites of cell-cell contact. We have also shown that these interactions occurred in both the cytoskeleton-associated and soluble pool of proteins, suggesting that these interactions have both adhesive and nonadhesive functions. Since plakoglobin was detected in the nucleus of plakoglobin-expressing SCC9 cells and since luciferase reporter assays have shown that *β*-catenin/Wnt signaling is not activated in SCC9 cells [65], these results together suggested that plakoglobin regulates gene expression in SCC9 cells independent of *β*-catenin. We are currently characterizing whether plakoglobin directly regulates Nm23 expression. Furthermore, we have also shown that plakoglobin interacts with the transcription factor p53 and regulates the expression of a number of p53 target genes (manuscript in preparation).

## 8. Concluding Remarks

Recent work has demonstrated that plakoglobin has novel roles in intracellular signaling and the regulation of gene expression, in addition to its previously well-established roles in cell-cell adhesion. Plakoglobin has emerged as a tumor/metastasis suppressor protein based on evidence from the great majority of the studies that have examined its signaling function. As more work focuses on the role of plakoglobin in tumorigenesis and metastasis, it is becoming clear that plakoglobin is a key, important player in these processes and consequently may be a useful therapeutic target in the treatment of cancer.

## References

[1] Peifer M, McCrea PD, Green KJ, Wieschaus E, Gumbiner BM (1992). The vertebrate adhesive junction proteins *β*-catenin and plakoglobin and the *Drosophila* segment polarity gene *armadillo* form a multigene family with similar properties. *Journal of Cell Biology*.

[2] Butz S, Stappert J, Weissig H (1992). Plakoglobin and *β*-catenin: distinct but closely related. *Science*.

[3] Ben-Ze’Ev A, Geiger B (1998). Differential molecular interactions of *β*-catenin and plakoglobin in adhesion, signaling and cancer. *Current Opinion in Cell Biology*.

[4] Zhurinsky J, Shtutman M, Ben-Ze’ev A (2000). Plakoglobin and *β*-catenin: protein interactions, regulation and biological roles. *Journal of Cell Science*.

[5] MacDonald BT, Tamai K, He X (2009). Wnt/*β*-Catenin signaling: components, mechanisms, and diseases. *Developmental Cell*.

[6] Rao TP, Kühl M (2010). An updated overview on wnt signaling pathways: a prelude for more. *Circulation Research*.

[7] Verkaar F, Zaman GJR (2011). New avenues to target Wnt/*β*-catenin signaling. *Drug Discovery Today*.

[8] Simcha I, Geiger B, Yehuda-Levenberg S, Salomon D, Ben-Ze’ev A (1996). Suppression of tumorigenicity by plakoglobin: an augmenting effect of N-cadherin. *Journal of Cell Biology*.

[9] Parker HR, Li Z, Sheinin H, Lauzon G, Pasdar M (1998). Plakoglobin induces desmosome formation and epidermoid phenotype in N- cadherin-expressing squamous carcinoma cells deficient in plakoglobin and E- cadherin. *Cell Motility and the Cytoskeleton*.

[10] Charpentier E, Lavker RM, Acquista E, Cowin P (2000). Plakoglobin suppresses epithelial proliferation and hair growth in vivo. *Journal of Cell Biology*.

[11] Franke WW, Mueller H, Mittnacht S, Kapprell HP, Jorcano JL (1983). Significance of two desmosome plaque-associated polypeptides of molecular weights 75 000 and 83 000. *The EMBO Journal*.

[12] Cowin P, Kapprell HP, Franke WW (1986). Plakoglobin: a protein common to different kinds of intercellular adhering junctions. *Cell*.

[13] Korman NJ, Eyre RW, Klaus-Kovtun V, Stanley JR (1989). Demonstration of an adhering-junction molecule (plakoglobin) in the autoantigens of pemphigus foliaceus and pemphigus vulgaris. *The New England Journal of Medicine*.

[14] Vestweber D, Kemler R (1984). Some structural and functional aspects of the cell adhesion molecular uvomorulin. *Cell Differentiation*.

[15] Peyrieras N, Louvard D, Jacob F (1985). Characterization of antigens recognized by monoclonal and polyclonal antibodies directed against uvomorulin. *Proceedings of the National Academy of Sciences of the United States of America*.

[16] Ozawa M, Kemler R (1992). Molecular organization of the uvomorulin-catenin complex. *Journal of Cell Biology*.

[17] McCrea PD, Turck CW, Gumbiner B (1991). A homolog of the *armadillo* protein in *Drosophila* (plakoglobin) associated with E-cadherin. *Science*.

[18] Knudsen KA, Wheelock MJ (1992). Plakoglobin, or an 83-kD homologue distinct from *β*-catenin, interacts with E-cadherin and N-cadherin. *Journal of Cell Biology*.

[19] Piepenhagen PA, Nelson WJ (1993). Defining E-cadherin-associated protein complexes in epithelial cells: plakoglobin, *β*- and *γ*- catenin are distinct components. *Journal of Cell Science*.

[20] Pasdar M, Li Z, Chlumecky V (1995). Plakoglobin: kinetics of synthesis, phosphorylation, stability, and interactions with desmoglein and E-cadherin. *Cell Motility and the Cytoskeleton*.

[21] Meng W, Takeichi M (2009). Adherens junction: molecular architecture and regulation. *Cold Spring Harbor Perspectives in Biology*.

[22] Harris TJC, Tepass U (2010). Adherens junctions: from molecules to morphogenesis. *Nature Reviews Molecular Cell Biology*.

[23] Garrod D, Chidgey M (2008). Desmosome structure, composition and function. *Biochimica et Biophysica Acta*.

[24] Dusek RL, Attardi LD (2011). Desmosomes: new perpetrators in tumour suppression. *Nature Reviews Cancer*.

[25] Nagafuchi A, Shirayoshi Y, Okazaki K (1987). Transformation of cell adhesion properties by exogenously introduced E-cadherin cDNA. *Nature*.

[26] Nose A, Nagafuchi A, Takeichi M (1988). Expressed recombinant cadherins mediate cell sorting in model systems. *Cell*.

[27] Mege RM, Matsuzaki F, Gallin WJ, Goldberg JE, Cunningham BA, Edelman GM (1988). Construction of epithelioid sheets by transfection of mouse sarcoma cells with cDNAs for chicken cell adhesion molecules. *Proceedings of the National Academy of Sciences of the United States of America*.

[28] Behrens J, Mareel MM, Van Roy FM, Birchmeier W (1989). Dissecting tumor cell invasion: epithelial cells acquire invasive properties after the loss of uvomorulin-mediated cell-cell adhesion. *Journal of Cell Biology*.

[29] Frixen UH, Behrens J, Sachs M (1991). E-cadherin-mediated cell-cell adhesion prevents invasiveness of human carcinoma cells. *Journal of Cell Biology*.

[30] Vleminckx K, Vakaet L, Mareel M, Fiers W, Van Roy F (1991). Genetic manipulation of E-cadherin expression by epithelial tumor cells reveals an invasion suppressor role. *Cell*.

[31] Chen W, Obrink B (1991). Cell-cell contacts mediated by E-cadherin (uvomorulin) restrict invasive behavior of L-cells. *Journal of Cell Biology*.

[32] Shimoyama Y, Hirohashi S, Hirano S (1989). Cadherin cell-adhesion molecules in human epithelial tissues and carcinomas. *Cancer Research*.

[33] Shimoyama Y, Hirohashi S (1991). Expression of E- and P-cadherin in gastric carcinomas. *Cancer Research*.

[34] Schipper JH, Frixen UH, Behrens J, Unger A, Jahnke K, Birchmeier W (1991). E-cadherin expression in squamous cell carcinomas of head and neck: inverse correlation with tumor dedifferentiation and lymph node metastasis. *Cancer Research*.

[35] Navarro P, Gomez M, Pizarro A, Gamallo C, Quintanilla M, Cano A (1991). A role for the E-cadherin cell-cell adhesion molecule during tumor progression of mouse epidermal carcinogenesis. *Journal of Cell Biology*.

[36] Mareel MM, Behrens J, Birchmeier W (1991). Down-regulation of E-cadherin expression in Madin Darby canine kidney (MDCK) cells inside tumors of nude mice. *International Journal of Cancer*.

[37] Gamallo C, Palacios J, Suarez A (1993). Correlation of E-cadherin expression with differentiation grade and histological type in breast carcinoma. *American Journal of Pathology*.

[38] Navarro P, Lozano E, Cano A (1993). Expression of E- or P-cadherin is not sufficient to modify the morphology and the tumorigenic behavior of murine spindle carcinoma cells. Possible involvement of plakoglobin. *Journal of Cell Science*.

[39] Lewis JE, Wahl JK, Sass KM, Jensen PJ, Johnson KR, Wheelock MJ (1997). Cross-talk between adherens junctions and desmosomes depends on plakoglobin. *Journal of Cell Biology*.

[40] Kemler R, Babinet C, Eisen H, Jacob F (1977). Surface antigen in early differentiation. *Proceedings of the National Academy of Sciences of the United States of America*.

[41] Gumbiner B, Simons K (1986). A functional assay for proteins involved in establishing and epithelial occluding barrier: identification of a uvomorulin-like polypeptide. *Journal of Cell Biology*.

[42] Hennings H, Holbrook KA (1983). Calcium regulation of cell-cell contact and differentiation of epidermal cells in culture. An ultrastructural study. *Experimental Cell Research*.

[43] Gumbiner B, Stevenson B, Grimaldi A (1988). The role of the cell adhesion molecule uvomorulin in the formation and maintenance of the epithelial junctional complex. *Journal of Cell Biology*.

[44] Wheelock MJ, Jensen PJ (1992). Regulation of keratinocyte intercellular junction organization and epidermal morphogenesis by E-cadherin. *Journal of Cell Biology*.

[45] Lewis JE, Jensen PJ, Wheelock MJ (1994). Cadherin function is required for human keratinocytes to assemble desmosomes and stratify in response to calcium. *Journal of Investigative Dermatology*.

[46] Jensen PJ, Telegan B, Lavker RM, Wheelock MJ (1997). E-cadherin and P-cadherin have partially redundant roles in human epidermal stratification. *Cell and Tissue Research*.

[47] Amagai M, Fujimori T, Masunaga T (1995). Delayed assembly of desmosomes in keratinocytes with disrupted classic-cadherin-mediated cell adhesion by a dominant negative mutant. *Journal of Investigative Dermatology*.

[48] Ruiz P, Brinkmann V, Ledermann B (1996). Targeted mutation of plakoglobin in mice reveals essential functions of desmosomes in the embryonic heart. *Journal of Cell Biology*.

[49] Li Z, Gallin WJ, Lauzon G, Paadar M (1998). L-CAM expression induces fibroblast-epidermoid transition in squamous carcinoma cells and down-regulates the endogenous N-cadherin. *Journal of Cell Science*.

[50] Palka HL, Green KJ (1997). Roles of plakoglobin end domains in desmosome assembly. *Journal of Cell Science*.

[51] Acehan D, Petzold C, Gumper I (2008). Plakoglobin is required for effective intermediate filament anchorage to desmosomes. *Journal of Investigative Dermatology*.

[52] Gosavi P, Kundu ST, Khapare N, Sehgal L, Karkhanis MS, Dalal SN (2011). E-cadherin and plakoglobin recruit plakophilin3 to the cell border to initiate desmosome assembly. *Cellular and Molecular Life Sciences*.

[53] Kundu ST, Gosavi P, Khapare N (2008). Plakophilin3 downregulation leads to a decrease in cell adhesion and promotes metastasis. *International Journal of Cancer*.

[54] Ruiz P, Birchmeier W (1998). The plakoglobin knock-out mouse: a paradigm for the molecular analysis of cardiac cell junction formation. *Trends in Cardiovascular Medicine*.

[55] Bradley RS, Cowin P, Brown AMC (1993). Expression of Wnt-1 in PC12 cells results in modulation of plakoglobin and E-cadherin and increased cellular adhesion. *Journal of Cell Biology*.

[56] Karnovsky A, Klymkowsky MW (1995). Anterior axis duplication in Xenopus induced by the over-expression of the cadherin-binding protein plakoglobin. *Proceedings of the National Academy of Sciences of the United States of America*.

[57] Kolligs FT, Kolligs B, Hajra KM (2000). *γ*-Catenin is regulated by the APC tumor suppressor and its oncogenic activity is distinct from that of *β*-catenin. *Genes and Development*.

[58] Bommer GT, Jäger C, Dürr EM (2005). DRO1, a gene down-regulated by oncogenes, mediates growth inhibition in colon and pancreatic cancer cells. *Journal of Biological Chemistry*.

[59] Pan H, Gao F, Papageorgis P, Abdolmaleky HM, Faller DV, Thiagalingam S (2007). Aberrant activation of *γ*-catenin promotes genomic instability and oncogenic effects during tumor progression. *Cancer Biology and Therapy*.

[60] Morin PJ, Sparks AB, Korinek V (1997). Activation of *β*-catenin-Tcf signaling in colon cancer by mutations in *β*-catenin or APC. *Science*.

[61] Salomon D, Sacco PA, Roy SG (1997). Regulation of *β*-catenin levels and localization by overexpression of plakoglobin and inhibition of the ubiquitin-proteasome system. *Journal of Cell Biology*.

[62] Simcha I, Shtutman M, Salomon D (1998). Differential nuclear translocation and transactivation potential of *β*- Catenin and plakoglobin. *Journal of Cell Biology*.

[63] Zhurinsky J, Shtutman M, Ben-Ze’ev A (2000). Differential mechanisms of LEF/TCF family-dependent transcriptional activation by *β*-catenin and plakoglobin. *Molecular and Cellular Biology*.

[64] Williams BO, Barish GD, Klymkowsky MW, Varmus HE (2000). A comparative evaluation of *β*-catenin and plakoglobin signaling activity. *Oncogene*.

[65] Li L, Chapman K, Hu X, Wong A, Pasdar M (2007). Modulation of the oncogenic potential of *β*-catenin by the subcellular distribution of plakoglobin. *Molecular Carcinogenesis*.

[66] Teulière J, Faraldo MM, Shtutman M (2004). *β*-catenin-dependent and -independent effects of ΔN-plakoglobin on epidermal growth and differentiation. *Molecular and Cellular Biology*.

[67] Klymkowsky MW, Williams BO, Barish GD, Varmus HE, Vourgourakis YE (1999). Membrane-anchored plakoglobins have multiple mechanisms of action in Wnt signaling. *Molecular Biology of the Cell*.

[68] Shibata T, Gotoh M, Ochiai A, Hirohashi S (1994). Association of plakoglobin with APC, a tumor suppressor gene product, and its regulation by tyrosine phosphorylation. *Biochemical and Biophysical Research Communications*.

[69] Kodama S, Ikeda S, Asahara T, Kishida M, Kikuchi A (1999). Axin directly interacts with plakoglobin and regulates its stability. *Journal of Biological Chemistry*.

[70] Merriam JM, Rubenstein AB, Klymkowsky MW (1997). Cytoplasmically anchored plakoglobin induces a WNT-like phenotype in Xenopus. *Developmental Biology*.

[71] Ozawa M, Terada H, Pedraza C (1995). The fourth *armadillo* repeat of plakoglobin (*γ*-catenin) is required for its high affinity binding to the cytoplasmic domains of E-cadherin and desmosomal cadherin Dsg2, and the tumor suppressor APC protein. *Journal of Biochemistry*.

[72] Aberle H, Schwartz H, Hoschuetzky H, Kemler R (1996). Single amino acid substitutions in proteins of the *armadillo* gene family abolish their binding to *α*-catenin. *Journal of Biological Chemistry*.

[73] Witcher LL, Collins R, Puttagunta S (1996). Desmosomal cadherin binding domains of plakoglobin. *Journal of Biological Chemistry*.

[74] Troyanovsky RB, Chitaev NA, Troyanovsky SM (1996). Cadherin binding sites of plakoglobin: localization, specificity and role in targeting to adhering junctions. *Journal of Cell Science*.

[75] Kowalczyk AP, Bornslaeger EA, Borgwardt JE (1997). The amino-terminal domain of desmoplakin binds to plakoglobin and clusters desmosomal cadherin-plakoglobin complexes. *Journal of Cell Biology*.

[76] Hatzfeld M, Green KJ, Sauter H (2003). Targeting of p0071 to desmosomes and adherens junctions is mediated by different protein domains. *Journal of Cell Science*.

[77] Hakimelahi S, Parker HR, Gilchrist AJ (2000). Plakoglobin regulates the expression of the anti-apoptotic protein BCL-2. *Journal of Biological Chemistry*.

[78] Rosin-Arbesfeld R, Cliffe A, Brabletz T, Bienz M (2003). Nuclear export of the APC tumour suppressor controls *β*-catenin function in transcription. *The EMBO Journal*.

[79] He TC, Sparks AB, Rago C (1998). Identification of c-MYC as a target of the APC pathway. *Science*.

[80] Zhou C, Liu S, Zhou X (2005). Overexpression of human pituitary tumor transforming gene (hPTTG), is regulated by *β*-catenin /TCF pathway in human esophageal squamous cell carcinoma. *International Journal of Cancer*.

[81] ten Berge D, Koole W, Fuerer C, Fish M, Eroglu E, Nusse R (2008). Wnt signaling mediates self-organization and axis formation in embryoid bodies. *Cell Stem Cell*.

[82] Conacci-Sorrell ME, Ben-Yedidia T, Shtutman M, Feinstein E, Einat P, Ben-Ze’ev A (2002). Nr-CAM is a target gene of the *β*-catenin/LEF-1 pathway in melanoma and colon cancer and its expression enhances motility and confers tumorigenesis. *Genes and Development*.

[83] Maeda O, Usami N, Kondo M (2004). Plakoglobin (*γ*-catenin) has TCF/LEF family-dependent transcriptional activity in *β*-catenin-deficient cell line. *Oncogene*.

[84] Shimizu M, Fukunaga Y, Ikenouchi J, Nagafuchi A (2008). Defining the roles of *β*-catenin and plakoglobin in LEF/T-cell factor-dependent transcription using *β*-catenin/plakoglobin-null F9 cells. *Molecular and Cellular Biology*.

[85] Kim YM, Ma H, Oehler VG (2011). The Gamma catenin/CBP complex maintains survivin transcription in *β*-catenin deficient/depleted cancer cells. *Current Cancer Drug Targets*.

[86] Winn RA, Bremnes RM, Bemis L (2002). *γ*-catenin expression is reduced or absent in a subset of human lung cancers and re-expression inhibits transformed cell growth. *Oncogene*.

[87] Pötter E, Braun S, Lehmann U, Brabant G (2001). Molecular cloning of a functional promoter of the human plakoglobin gene. *European Journal of Endocrinology*.

[88] Husmark J, Heldin NE, Nilsson M (1999). N-cadherin-mediated adhesion and aberrant catenin expression in anaplastic thyroid-carcinoma cell lines. *International Journal of Cancer*.

[89] Shiina H, Breault JE, Basset WW (2005). Functional loss of the *γ*-catenin gene through epigenetic and genetic pathways in human prosthate cancer. *Cancer Research*.

[90] Canes D, Chiang GJ, Billmeyer BR (2005). Histone deacetylase inhibitors upregulate plakoglobin expression in bladder carcinoma cells and display antineoplastic activity in vitro and in vivo. *International Journal of Cancer*.

[91] Rahnama F, Shafiei F, Gluckman PD, Mitchell MD, Lobie PE (2006). Epigenetic regulation of human trophoblastic cell migration and invasion. *Endocrinology*.

[92] Shafiei F, Rahnama F, Pawella L, Mitchell MD, Gluckman PD, Lobie PE (2008). DNMT3A and DNMT3B mediate autocrine hGH repression of plakoglobin gene transcription and consequent phenotypic conversion of mammary carcinoma cells. *Oncogene*.

[93] Dusek RL, Godsel LM, Chen F (2007). Plakoglobin deficiency protects keratinocytes from apoptosis. *Journal of Investigative Dermatology*.

[94] Nagashima H, Okada M, Hidai C, Hosoda S, Kasanuki H, Kawana M (1997). The role of cadherin-catenin-cytoskeleton complex in angiogenesis: antisense oligonucleotide of plakoglobin promotes angiogenesis in vitro, and protein kinase C (PKC) enhances angiogenesis through the plakoglobin signaling pathway. *Heart and Vessels*.

[95] Mukhina S, Mertani HC, Guo K, Lee KO, Gluckman PD, Lobie PE (2004). Phenotypic conversion of human mammary carcinoma cells by autocrine human growth hormone. *Proceedings of the National Academy of Sciences of the United States of America*.

[96] Rieger-Christ KM, Ng L, Hanley RS (2005). Restoration of plakoglobin expression in bladder carcinoma cell lines suppresses cell migration and tumorigenic potential. *British Journal of Cancer*.

[97] Yin T, Getsios S, Caldelari R (2005). Plakoglobin supresses keratinocyte motility through both cell-cell adhesion-dependent and -independent mechanisms. *Proceedings of the National Academy of Sciences of the United States of America*.

[98] Todorović V, Desai BV, Patterson MJS (2010). Plakoglobin regulates cell motility through Rho- and fibronectin-dependent Src signaling. *Journal of Cell Science*.

[99] Williamson L, Raess NA, Caldelari R (2006). Pemphigus vulgaris identifies plakoglobin as key suppressor of c-Myc in the skin. *The EMBO Journal*.

[100] Miravet S, Piedra J, Miró F, Itarte E, De Herreros AG, Duñach M (2002). The transcriptional factor Tcf-4 contains different binding sites for *β*-catenin and plakoglobin. *Journal of Biological Chemistry*.

[101] Shtutman M, Zhurinsky J, Oren M, Levina E, Ben-Ze’ev A (2002). PML is a target gene of *β*-catenin and plakoglobin, and coactivates *β*-catenin-mediated transcription. *Cancer Research*.

[102] Aberle H, Bierkamp C, Torchard D (1995). The human plakoglobin gene localizes on chromosome 17q21 and is subjected to loss of heterozygosity in breast and ovarian cancers. *Proceedings of the National Academy of Sciences of the United States of America*.

[103] Syrigos KN, Harrington K, Waxman J, Krausz T, Pignatelli M (1998). Altered *γ*-catenin expression correlates with poor survival in patients with bladder cancer. *Journal of Urology*.

[104] Clairotte A, Lascombe I, Fauconnet S (2006). Expression of E-cadherin and *α*-, *β*-, *γ*-catenins in patients with bladder cancer: identification of *γ*-catenin as a new prognostic marker of neoplastic progression in T1 superficial urothelial tumors. *American Journal of Clinical Pathology*.

[105] Tziortzioti V, Ruebel KH, Kuroki T, Jin L, Scheithauer BW, Lloyd RV (2001). Analysis of *β*-catenin mutations and *α*-, *β*-, and *γ*-catenin expression in normal and neoplastic human pituitary tissues. *Endocrine Pathology*.

[106] Tada H, Hatoko M, Tanaka A, Kuwahara M, Muramatsu T (2000). Expression of desmoglein I and plakoglobin in skin carcinomas. *Journal of Cutaneous Pathology*.

[107] Muzio LL, Staibano S, Pannone G (1999). Beta- and gamma-catenin expression in oral squamous cell carcinomas. *Anticancer Research*.

[108] Morita N, Uemura H, Tsumatani K (1999). E-cadherin and *α*-, *β*- and *γ*-catenin expression in prostate cancers: correlation with tumour invasion. *British Journal of Cancer*.

[109] Ueda G, Sunakawa H, Nakamori K (2006). Aberrant expression of *β*- and *γ*-catenin is an independent prognostic marker in oral squamous cell carcinoma. *International Journal of Oral and Maxillofacial Surgery*.

[110] Depondt J, Shabana AH, Florescu-Zorila S, Gehanno P, Forest N (1999). Down-regulation of desmosomal molecules in oral and pharyngeal squamous cell carcinomas as a marker for tumour growth and distant metastasis. *European Journal of Oral Sciences*.

[111] Bremnes RM, Veve R, Gabrielson E (2002). High-throughput tissue microarray analysis used to evaluate biology and prognostic significance of the E-cadherin pathway in non-small-cell lung cancer. *Journal of Clinical Oncology*.

[112] Sanders DSA, Blessing K, Hassan GAR, Bruton R, Marsden JR, Jankowski J (1999). Alterations in cadherin and catenin expression during the biological progression of melanocytic tumours. *Journal of Clinical Pathology*.

[113] Cerrato A, Fulciniti F, Avallone A, Benincasa G, Palombini L, Grieco M (1998). Beta- and gamma-catenin expression in thyroid carcinomas. *Journal of Pathology*.

[114] Papagerakis S, Shabana AH, Depondt J, Pibouin L, Blin-Wakkach C, Berdal A (2004). Altered Plakoglobin Expression at mRNA and Protein Levels Correlates with Clinical Outcome in Patients with Oropharynx Squamous Carcinomas. *Human Pathology*.

[115] Lifschitz-Mercer B, Amitai R, Maymon BBS (2001). Nuclear localization of *β*-catenin and plakoglobin in primary and metastatic human colonic carcinomas, colonic adenomas, and normal colon. *International Journal of Surgical Pathology*.

[116] Lin YC, Wu MY, Li DR, Wu XY, Zheng RM (2004). Prognostic and clinicopathological features of E-cadherin, *α* -catenin, *β*-catenin, *γ*-catenin and cyclin D1 expression in human esophageal squamous cell carcinoma. *World Journal of Gastroenterology*.

[117] Buchner A, Oberneder R, Riesenberg R, Keiditsch E, Hofstetter A (1998). Expression of plakoglobin in renal cell carcinoma. *Anticancer Research*.

[118] Basta-Jovanovic G, Gvozdenovic E, Dimitrijević I (2008). Immunohistochemical analysis of gamma catenin in Wilms' tumors. *Fetal and Pediatric Pathology*.

[119] Kanazawa Y, Ueda Y, Shimasaki M (2008). Down-regulation of Plakoglobin in soft tissue sarcoma is associated with a higher risk of pulmonary metastasis. *Anticancer Research*.

[120] Kim YT, Choi EK, Kim JW, Kim DK, Kim SH, Yang WI (2002). Expression of E-cadherin and *α*-, *β*-, *γ*-catenin proteins in endometrial carcinoma. *Yonsei Medical Journal*.

[121] Misaki K, Marukawa K, Hayashi Y (2005). Correlation of *γ*-catenin expression with good prognosis in medulloblastomas. *Journal of Neurosurgery*.

[122] Närkiö-Mäkelä M, Pukkila M, Lagerstedt E (2009). Reduced *γ*-catenin expression and poor survival in oral squamous cell carcinoma. *Archives of Otolaryngology—Head and Neck Surgery*.

[123] Baumgart E, Cohen MS, Neto BS (2007). Identification and prognostic significance of an epithelial-mesenchymal transition expression profile in human bladder tumors. *Clinical Cancer Research*.

[124] Aktary Z, Chapman K, Lam L (2010). Plakoglobin interacts with and increases the protein levels of metastasis suppressor Nm23-H2 and regulates the expression of Nm23-H1. *Oncogene*.

[125] Steeg PS, Horak CE, Miller KD (2008). Clinical-translational approaches to the Nm23-H1 metastasis Suppressor. *Clinical Cancer Research*.

[126] Marshall JC, Collins J, Marino N, Steeg P (2010). The Nm23-H1 metastasis suppressor as a translational target. *European Journal of Cancer*.

